# Supplementary material for: Genome-wide analysis of DNA G-quadruplex motifs across 37 species provides insights into G4 evolution
Source: Commun Biol. 2021 Jan 22;4:98. doi: 10.1038/s42003-020-01643-4 (PMC7822830; doi:10.1038/s42003-020-01643-4)
Supplement: Supplementary file 2 — Supplementary Information [file 42003_2020_1643_MOESM2_ESM.pdf]

SUPPLEMENTARY INFORMATION

Genome-wide analysis of DNA G-quadruplex motifs across 37 species provides insights into G4 evolution

Feng Wu[1,6], Kangkang Niu[1, 6], Yong Cui[1, 6], Cencen Li[2], Mo Lyu[1], Yandong Ren[3], Yanfei Chen[1], Huimin Deng[1], Lihua Huang[1], Sichun Zheng[1], Lin Liu[1], Jian Wang[4], Qisheng Song[5,*], Hui Xiang[1,*] and Qili Feng[1,*]

[1] Guangdong Provincial Key Laboratory of Insect Developmental Biology and Applied Technology, Guangzhou Key Laboratory of Insect Development Regulation and Application Research, Institute of Insect Science and Technology, School of Life Sciences, South China Normal University, Guangzhou, 510631, China.
[2] College of Life Sciences, Xinyang Normal University, Xinyang, 464000, China.
[3] Center for Ecological and Environmental Sciences, Northwestern Polytechnical University, Xi'an, 710129, China.
[4] Department of Entomology, University of Maryland, College Park, MD 20742, USA
[5] Division of Plant Sciences, University of Missouri, Columbia, MO 65211, USA

[6]These authors contributed equally.

* To whom correspondence should be addressed. Correspondence may also be addressed to Qili Feng, Tel: +86 20 85215291; Fax: +86 20 85215291; Email: qlfeng@scnu.edu.cn; Hui Xiang, Tel: +86 20 85210024; Fax: +86 20 85215291; Email: xiang_shine@foxmail.com and Qisheng Song, Tel: +1 573 8829798; Fax: +1 573 8821469; Email: songq@missouri.edu

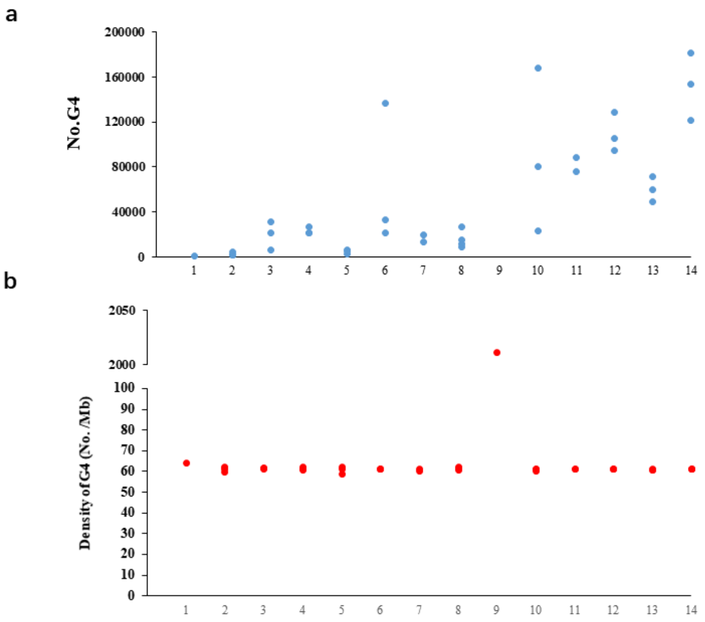

Supplementary Fig. 1 G4 motifs in the random sequences with same sizes to the 37 species of the 14 representative evolutionary nodes. a. G4 number. b. G4 density.

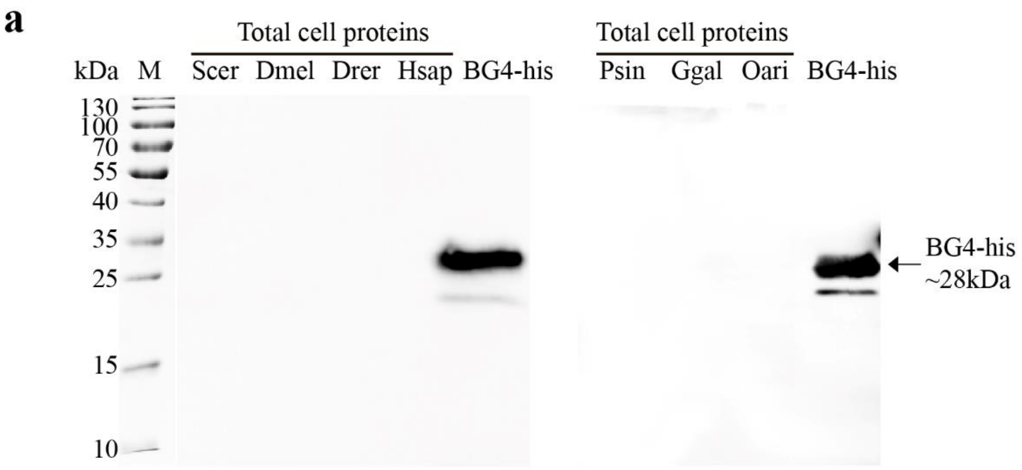

Supplementary Fig. 2 Western blot analyses confirming that the anti-His antibody could specifically recognize BG4-His but could not recognize other proteins in the cells.

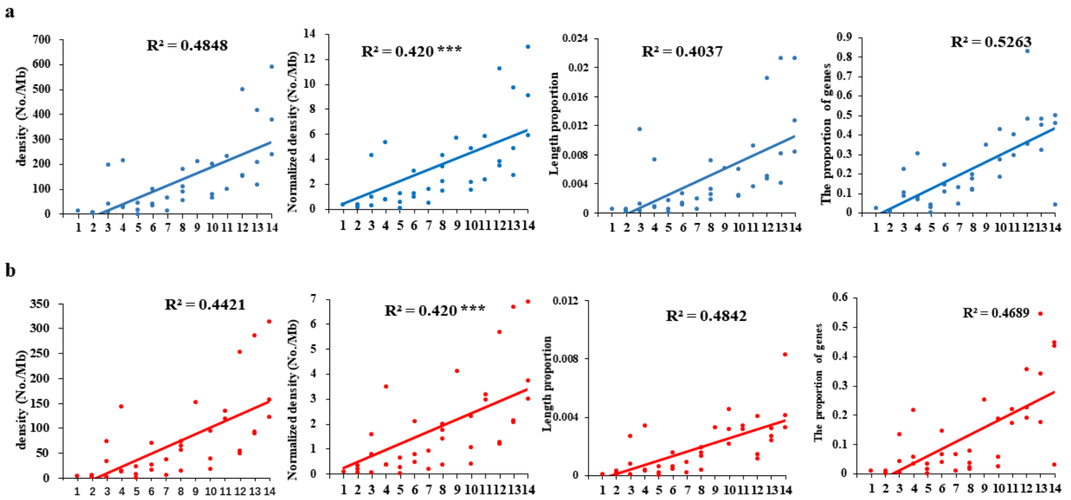

Supplementary Fig 3. Genomic landscape (density, normalized density, length ratio of motifs in the genomes, and the proportion of the genes bearing the motifs in the upstream 2 kb region) of $(G/C)_3L_{1\text{-}12}$ (a) and $G/C)_3L_{1\text{-}7}$ (b) motifs in the 37 species. R2: goodness of fit of the trend lines. *, p<0.05; **, p<0.01; and ***, p<0.001 by F test. n= 37 biologically independent samples.

Supplementary Table 1 Genome information and statistics of G4 motifs in the genomes of the 37 species

Category	Species	Genome size(bp)	GC content(%)	No. Genes	G4 count	Normalized G4 count	G4 Density(No./Mb)	Normalized G4 density(No./Mb)	Total G4 length	$(G/C)_3L_{1-7}$ count	Normalized $(G/C)_3L_{1-7}$ count	$(G/C)_3L_{1-7}$ density	Normalized $(G/C)_3L_{1-7}$ density	Total $(G/C)_3L_{1-7}$ length	$(G/C)_3L_{1-12}$ count	Normalized $(G/C)_3L_{1-12}$ count	$(G/C)_3L_{1-12}$ density	Normalized $(G/C)_3L_{1-12}$ density	Total $(G/C)_3L_{1-12}$ length
Fungi	*Saccharomyces cerevisiae*	12157105	38.148	6,692	4705	123.336	405.816	10.638	83856	38	0.996	3.278	0.086	956	143	3.749	12.334	0.323	6178
Protozoa	*Plasmodium reichenowi*	24055504	19.258	5,630	2400	124.623	104.616	5.432	60949	144	7.477	6.277	0.326	6779	166	8.620	7.236	0.376	13574
	Paramecium tetraurelia	72094543	28.051	39,642	8527	303.979	124.021	4.421	173563	139	4.955	2.022	0.072	12193	293	10.445	4.262	0.152	17600
	Dictyostelium discoideum	34134454	22.432	13,243	12518	558.031	384.540	17.142	225113	139	6.196	4.270	0.190	3350	232	10.342	7.127	0.318	6979
Platyhelminthes	*Schistosoma mansoni*	364541798	35.208	7,544	68679	1950.661	197.550	5.611	1202175	781	22.182	2.246	0.064	21085	3762	106.851	10.821	0.307	117326
	Macrostomum lignano	536655498	46.093	49,027	1538682	33382.182	3006.445	65.226	35865996	37477	813.075	73.227	1.589	1443464	101002	2191.270	197.349	4.282	6179448
	Echinococcus granulosus	98695753	43.359	11,319	112264	2589.179	1192.729	27.508	2012044	3198	73.756	33.977	0.784	78597	4066	93.775	43.199	0.996	128867
Coelenterata	*Nematostella vectensis*	356613585	40.635	24,808	462104	11372.027	1358.757	33.438	10238516	48544	1194.631	142.737	3.513	1211288	73884	1818.229	217.246	5.346	2611698
	Acropora digitifera	357255219	40.732	32,469	183885	4514.563	539.719	13.251	3429280	5199	127.641	15.260	0.375	132716	10484	257.393	30.771	0.755	338783
	Hydra vulgaris	454579533	34.439	22,980	207130	6014.425	477.786	13.873	3623462	5428	157.613	12.521	0.364	134913	11547	335.290	26.635	0.773	378936
Nematoda	*Caenorhabditis elegans*	100286401	35.440	35,827	60994	1721.062	637.742	17.995	1211504	2227	62.839	23.285	0.657	57917	4291	121.079	44.866	1.266	177685
	Strongyloides ratti	43150242	21.427	12,449	3518	164.183	85.489	3.990	63476	17	0.793	0.413	0.019	564	39	1.820	0.948	0.044	1769
	Brugia malayi	94062919	29.587	13,571	16321	551.636	181.940	6.149	330960	699	23.626	7.792	0.263	19455	1407	47.555	15.685	0.530	49866

Mollusca	*Lottia gigantea*	359505668	33.279	23,361	120565	3622.858	351.654	10.567	2431111	9120	274.047	26.600	0.799	228368	14386	432.285	41.960	1.261	475742
	Octopus bimaculoides	2338188782	36.041	33,610	1599129	44370.285	717.140	19.898	30293985	37392	1037.498	16.769	0.465	994826	76099	2111.483	34.127	0.947	2725830
	Crassostrea gigas	557717710	33.425	26,104	383419	11471.083	720.874	21.567	7253782	37679	1127.276	70.841	2.119	868336	54490	1630.225	102.448	3.065	1486402
Annelida	*Capitella teleta*	333283208	40.358	32,192	224940	5573.596	707.706	17.536	4270435	11947	296.024	37.588	0.931	295211	20648	511.619	64.963	1.610	659899
	Helobdella robusta	235376169	32.820	23,434	123770	3771.139	551.382	16.800	2615233	1493	45.490	6.651	0.203	47373	3392	103.351	15.111	0.460	131168
Arthropoda	*Apis mellifera*	250270657	32.696	13,287	275908	8438.524	1155.991	35.355	5456071	15503	474.152	64.954	1.987	384884	26538	811.653	111.188	3.401	825000
	Bombyx mori	453421119	38.317	14,623	411423	10737.431	951.452	24.831	6959101	6278	163.845	14.518	0.379	174338	23769	620.330	54.968	1.435	859614
	Drosophila melanogaster	143726002	42.006	14,151	209804	4994.598	1530.659	36.439	4528697	10182	242.393	74.284	1.768	277585	24814	590.723	181.034	4.310	1037348
	Danaus pulex	197206209	40.765	30,590	200618	4921.339	1066.717	26.168	577360	10728	263.167	57.042	1.399	255441	17007	417.197	90.429	2.218	515060
Echinodermata	*Strongylocentrotus purpuratus*	936564995	37.012	29,057	1323932	35769.958	1482.271	40.048	26639899	136075	3676.471	152.349	4.116	3053674	189456	5118.717	212.114	5.731	5700458
Fish	*Latimeria chalumnae*	2860591921	41.151	19,569	4674336	113589.852	1713.420	41.637	20663376	258792	6288.847	94.863	2.305	6193097	546547	13281.518	200.342	4.868	17225062
	Branchiostoma floridae	402213743	42.099	28,627	161412	3834.061	420.803	9.995	19179329	6678	158.624	17.410	0.414	1266308	25228	599.247	65.770	1.562	916002
	Danio rerio	1371719383	36.640	26,801	1061780	28978.704	811.651	22.152	70742161	50974	1391.211	38.966	1.063	6191298	103259	2818.203	78.934	2.154	3353374
Amphibian	*Xenopus tropicalis*	1511735326	40.063	18,496	1907074	47601.302	1322.792	33.017	43271594	171278	4275.165	118.803	2.965	4822597	335441	8372.737	232.670	5.808	14000561
	Nanorana parkeri	1297236036	42.422	21,416	3209691	75660.612	2594.443	61.158	58731184	167281	3943.240	135.216	3.187	4393760	123420	2909.325	99.762	2.352	4746960
Reptilia	*Anolis carolinensis*	1799143587	40.319	9,086	1598056	39635.108	931.378	23.100	32018066	83641	2074.471	48.748	1.209	2019034	265830	6593.136	154.931	3.843	8477996
	Pelodiscus sinensis	2202483752	44.410	18,189	4494044	101194.416	2139.560	48.178	105659088	530115	11936.953	252.381	5.683	8890451	1049290	23627.562	499.554	11.249	40741489

	Alligator sinensis	1619988496	43.260	22,381	4994172	115445.492	3232.596	74.724	96083248	85233	1970.229	55.169	1.275	2309308	233853	5405.698	151.367	3.499	8231961
Aves	*Gallus gallus*	1230258557	42.850	15,589	3107195	72513.753	2648.330	61.805	62966332	336212	7846.303	286.561	6.688	3951627	487757	11382.965	415.726	9.702	26148201
	Pseudopodoces humilis	844939770	43.127	16,586	1061678	24617.207	1317.550	30.550	24573536	72055	1670.745	89.421	2.073	2266233	169186	3922.928	209.961	4.868	6855902
	Struthio camelus	1011267964	43.120	16,646	1057265	24519.043	1096.270	25.424	27887498	89060	2065.391	92.346	2.142	2406508	112816	2616.317	116.978	2.713	4151884
Mammalia	*Ornithorhynchus anatinus*	2073148626	45.499	33,610	2110195	46378.734	1067.314	23.458	64103134	619883	13624.044	313.530	6.891	17188827	1165798	25622.388	589.648	12.960	44161831
	Ovis aries	2619054388	41.829	20,195	5532861	132273.327	2215.160	52.957	121674387	391424	9357.624	156.712	3.746	10737494	948705	22680.329	379.828	9.080	33233821
	Homo sapiens	3099750720	40.863	20,449	7356494	180028.241	2488.537	60.899	146013944	362817	8878.854	122.733	3.004	10142187	711650	17415.493	240.735	5.891	26023695

Supplementary Table 2 Enrichment of the $(G/C)_3L_{1\text{-}7}$ G4 motif-bearing genes in the function of transcription factor activity in 19 representative species

Phylogenetic group	Species	GO ID	Description	Genes	*P* value	Corrected p value
**Fungi**	***Saccharomyces cerevisiae***					
		GO:0000982	Transcription factor activity, RNA polymerase II core promoter proximal region sequence-specific binding	1	0.2332	0.3887
		GO:0003700	Transcription factor activity, sequence-specific DNA binding	2	0.3611	0.5015
**Protozoa**	***Dictyostelium discoideum***					
		GO:0003700	Transcription factor activity, sequence-specific DNA binding	2	0.1909	0.3858
**Platyhelminthes**	***Schistosoma mansoni***					
		GO:0000981	RNA polymerase II transcription factor activity, sequence-specific DNA binding	1	0.2296	0.6561
**Coelenterata**	***Nematostella vectensis***					
		GO:0000991	Transcription factor activity, core RNA polymerase II binding	1	0.2235	0.6667
		GO:0000982	Transcription factor activity, RNA polymerase II core promoter proximal region sequence-specific binding	4	0.4404	0.8171
		GO:0004879	RNA polymerase II transcription factor activity, ligand-activated sequence-specific DNA binding	1	0.8974	0.9844
		GO:0000981	RNA polymerase II transcription factor activity, sequence-specific DNA binding	21	0.9064	0.9874
		GO:0001076	Transcription factor activity, RNA polymerase II transcription factor binding	4	0.9153	0.9880
		GO:0001071	Nucleic acid binding transcription factor activity	59	0.9441	0.9939
		GO:0003700	Transcription factor activity, sequence-specific DNA binding	59	0.9441	0.9939
		GO:0000988	Transcription factor activity, protein binding	13	0.9500	0.9939
		GO:0000989	Transcription factor activity, transcription factor binding	12	0.9664	0.9992
**Nematoda**	***Caenorhabditis elegans***					
		GO:0000989	Transcription factor activity, transcription factor binding	4	0.3828	0.7078
		GO:0000988	Transcription factor activity, protein binding	4	0.3916	0.7117
		GO:0001071	Nucleic acid binding transcription factor activity	17	0.7549	0.8965
		GO:0003700	Transcription factor activity, sequence-specific DNA binding	17	0.7549	0.8965
		GO:0000981	RNA polymerase II transcription factor activity, sequence-specific DNA binding	3	0.8135	0.9163
**Nematoda**	***Brugia malayi***					
		GO:0003700	Transcription factor activity, sequence-specific DNA binding	5	0.1349	0.3654
**Mollusca**	***Lottia gigantea***					
		GO:0000988	Transcription factor activity, protein binding	4	0.2947	0.5849

		GO:0000989	Transcription factor activity, transcription factor binding	4	0.2947	0.5849
		GO:0001076	Transcription factor activity, RNA polymerase II transcription factor binding	1	0.7228	0.8462
		GO:0001071	Nucleic acid binding transcription factor activity	5	0.9988	1.0000
		GO:0003700	Transcription factor activity, sequence-specific DNA binding	5	0.9988	1.0000
**Mollusca**	***Crassostrea gigas***					
		GO:0001026	TFIIIB-type transcription factor activity	1	0.2749	0.6370
		GO:0000989	Transcription factor activity, transcription factor binding	11	0.6224	0.8232
		GO:0001076	Transcription factor activity, RNA polymerase II transcription factor binding	3	0.7399	0.8868
		GO:0004879	RNA polymerase II transcription factor activity, ligand-activated sequence-specific DNA binding	1	0.7997	0.9114
		GO:0003700	Transcription factor activity, sequence-specific DNA binding	23	0.9900	0.9932
**Annelida**	***Capitella teleta***					
		GO:0001071	Nucleic acid binding transcription factor activity	30	0.0054	0.3546
		GO:0003700	Transcription factor activity, sequence-specific DNA binding	30	0.0054	0.3546
		GO:0000981	RNA polymerase II transcription factor activity, sequence-specific DNA binding	5	0.0074	0.3700
		GO:0004879	RNA polymerase II transcription factor activity, ligand-activated sequence-specific DNA binding	4	0.0082	0.3700
		GO:0098531	Transcription factor activity, direct ligand regulated sequence-specific DNA binding	4	0.0082	0.3700
		GO:0001187	Transcription factor activity, RNA polymerase I CORE element binding transcription factor recruiting	1	0.0718	0.5055
		GO:0000989	Transcription factor activity, transcription factor binding	3	0.6362	0.8477
**Annelida**	***Helobdella robusta***					
		GO:0003700	Transcription factor activity, sequence-specific DNA binding	6	0.0977	0.5975
**Arthropoda**	***Apis mellifera***					
		**GO:0001071**	**Nucleic acid binding transcription factor activity**	**27**	**0.0000**	**0.0000**
		**GO:0003700**	**Transcription factor activity, sequence-specific DNA binding**	**27**	**0.0000**	**0.0000**
		GO:0000981	RNA polymerase II transcription factor activity, sequence-specific DNA binding	3	0.0135	0.1185
		GO:0004879	RNA polymerase II transcription factor activity, ligand-activated sequence-specific DNA binding	1	0.2685	0.5885
		GO:0000989	Transcription factor activity, transcription factor binding	1	0.8092	1.0000
**Arthropoda**	***Bombyx mori***					
		GO:0001071	Nucleic acid binding transcription factor activity	10	0.0083	0.2479

		GO:0003700	Transcription factor activity, sequence-specific DNA binding	10	0.0083	0.2479
**Arthropoda**	***Drosophila melanogaster***					
		**GO:0001071**	**Nucleic acid binding transcription factor activity**	**70**	**0.0000**	**0.0000**
		GO:0000988	Protein binding transcription factor activity	9	0.6682	1.0000
**Echinodermata**	***Strongylocentrotus purpuratus***					
		GO:0001075	Transcription factor activity, RNA polymerase II core promoter sequence-specific binding involved in preinitiation complex assembly	2	0.0653	0.6149
		GO:0000991	Transcription factor activity, core RNA polymerase II binding	1	0.2556	0.7064
		GO:0001076	Transcription factor activity, RNA polymerase II transcription factor binding	11	0.4485	0.7935
		GO:0000982	Transcription factor activity, RNA polymerase II core promoter proximal region sequence-specific binding	5	0.5035	0.8358
		GO:0000988	Transcription factor activity, protein binding	30	0.5275	0.8583
		GO:0000989	Transcription factor activity, transcription factor binding	29	0.5906	0.8610
		GO:0000981	RNA polymerase II transcription factor activity, sequence-specific DNA binding	38	0.6813	0.8984
		GO:0001083	Transcription factor activity, RNA polymerase II basal transcription factor binding	1	0.6930	0.8984
		GO:0001129	RNA polymerase II transcription factor activity, TBP-class protein binding, involved in preinitiation complex assembly	1	0.6930	0.8984
		GO:0001132	RNA polymerase II transcription factor activity, TBP-class protein binding	1	0.6930	0.8984
		GO:0001071	Nucleic acid binding transcription factor activity	98	0.6962	0.9001
		GO:0003700	Transcription factor activity, sequence-specific DNA binding	98	0.6962	0.9001
		GO:0004879	RNA polymerase II transcription factor activity, ligand-activated sequence-specific DNA binding	2	0.9786	1.0000
		GO:0098531	Transcription factor activity, direct ligand regulated sequence-specific DNA binding	2	0.9786	1.0000
**Fish**	***Danio rerio***					
		GO:0001071	Nucleic acid binding transcription factor activity	47	0.1072	0.8044
		GO:0000988	Protein binding transcription factor activity	9	0.6281	1.0000
**Amphibian**	***Xenopus tropicalis***					
		**GO:0001071**	**Nucleic acid binding transcription factor activity**	**182**	**0.0000**	**0.0013**
		**GO:0003700**	**Transcription factor activity, sequence-specific DNA binding**	**182**	**0.0000**	**0.0013**
		GO:0000981	RNA polymerase II transcription factor activity, sequence-specific DNA binding	80	0.0001	0.0552
		GO:0004879	RNA polymerase II transcription factor activity, ligand-activated sequence-specific DNA binding	12	0.0220	0.6872

		GO:0098531	Transcription factor activity, direct ligand regulated sequence-specific DNA binding	12	0.0220	0.6872
		GO:0000982	Transcription factor activity, RNA polymerase II core promoter proximal region sequence-specific binding	15	0.0403	0.6872
		GO:0000983	Transcription factor activity, RNA polymerase II core promoter sequence-specific	4	0.0481	0.6872
		GO:0001075	Transcription factor activity, RNA polymerase II core promoter sequence-specific binding involved in preinitiation complex assembly	4	0.0481	0.6872
		GO:0003705	Transcription factor activity, RNA polymerase II distal enhancer sequence-specific binding	2	0.4852	0.7988
		GO:0000989	Transcription factor activity, transcription factor binding	33	0.6795	0.8742
		GO:0000988	Transcription factor activity, protein binding	33	0.7090	0.8742
		GO:0001076	transcription factor activity, RNA polymerase II transcription factor binding	3	0.9983	1.0000
**Aves**	***Gallus gallus***					
		GO:0000988	Protein binding transcription factor activity	121	0.2329	0.5060
		GO:0001071	Nucleic acid binding transcription factor activity	233	0.4673	0.7181
**Mammalia**	***Ovis aries***					
		**GO:0001071**	**Nucleic acid binding transcription factor activity**	**394**	**0.0000**	**0.0000**
		**GO:0003700**	**Transcription factor activity, sequence-specific DNA binding**	**394**	**0.0000**	**0.0000**
		**GO:0000981**	**RNA polymerase II transcription factor activity, sequence-specific DNA binding**	**230**	**0.0000**	**0.0000**
		**GO:0000982**	**Transcription factor activity, RNA polymerase II core promoter proximal region sequence-specific binding**	**145**	**0.0000**	**0.0000**
		GO:0003705	Transcription factor activity, RNA polymerase II distal enhancer sequence-specific binding	34	0.0036	0.1441
		GO:0001076	Transcription factor activity, RNA polymerase II transcription factor binding	55	0.0042	0.1583
		GO:0000988	Transcription factor activity, protein binding	160	0.0066	0.2095
		GO:0000989	Transcription factor activity, transcription factor binding	157	0.0108	0.2848
		GO:0004879	RNA polymerase II transcription factor activity, ligand-activated sequence-specific DNA binding	18	0.1558	0.7389
		GO:0098531	Transcription factor activity, direct ligand regulated sequence-specific DNA binding	18	0.1558	0.7389
		GO:0000983	Transcription factor activity, RNA polymerase II core promoter sequence-specific	6	0.3138	0.7389
		GO:0001134	Transcription factor activity, transcription factor recruiting	2	0.3941	0.7389
		GO:0001010	Transcription factor activity, sequence-specific DNA binding transcription factor recruiting	1	0.4289	0.7389
		GO:0001087	Transcription factor activity, TFIIB-class binding	1	0.4289	0.7389

		GO:0001135	Transcription factor activity, RNA polymerase II transcription factor recruiting	1	0.4289	0.7389
		GO:0038051	Glucocorticoid-activated RNA polymerase II transcription factor binding transcription factor activity	1	0.4289	0.7389
		GO:0038052	RNA polymerase II transcription factor activity, estrogen-activated sequence-specific DNA binding	1	0.6739	0.8616
**Mammalia**	***Homo sapiens***					
		**GO:0001071**	**Nucleic acid binding transcription factor activity**	**666**	**0.0000**	**0.0000**
		**GO:0000988**	**Protein binding transcription factor activity**	**332**	**0.0000**	**0.0000**

Supplementary Table 3 The (G/C)3L1-7 motif in the upstream 2kb of pair-wise orthologous genes

Group	No. orthologs	No.$(G/C)_3L_{1-7}$ in uptream 2kb	No. Genes bearing $(G/C)_3L_{1-7}$ in upstream 2kb
D.mel-S.cer	2008	2	2
D.mel-P.rei	1467	0	0
D.mel-S.man	3972	11	11
D.mel-N.vec	5072	1903	1438
D.mel-C.ele	4467	158	150
D.mel-L.gig	5784	472	416
D.mel-C.tel	5871	611	542
D.mel-S.pur	5469	2024	1529
D.mel-D.rer	5795	337	296
D.mel-X.tro	5547	2229	1307
D.mel-Acar	5464	2539	1995
D.mel-G.gal	5450	7815	3103
D.mel-H.sap	5754	5442	2765

Supplementary Table 4 The links to download genome information of the species used in this study

Category	Species	Links to genome data
Fungi	*Saccharomyces cerevisiae*	http://fungi.ensembl.org/Saccharomyces_cerevisiae/Info/Index
Protozoa	*Plasmodium reichenowi*	http://protists.ensembl.org/Plasmodium_reichenowi_gca_001601855/Info/Index
	Paramecium tetraurelia	http://protists.ensembl.org/Paramecium_tetraurelia/Info/Index
	Dictyostelium discoideum	http://protists.ensembl.org/Dictyostelium_discoideum/Info/Index
Platyhelminthes	*Schistosoma mansoni*	http://metazoa.ensembl.org/Schistosoma_mansoni/Info/Index
	Macrostomum lignano	https://www.ncbi.nlm.nih.gov/genome/?term=Macrostomum_lignano
	Echinococcus granulosus	https://www.ncbi.nlm.nih.gov/genome/?term=Echinococcus_granulosus
Coelenterata	*Nematostella vectensis*	http://metazoa.ensembl.org/Nematostella_vectensis/Info/Index
	Acropora digitifera	https://www.ncbi.nlm.nih.gov/genome/?term=Acropora_digitifera
	Hydra vulgaris	https://www.ncbi.nlm.nih.gov/genome/?term=Hydra_vulgaris
Nematoda	*Caenorhabditis elegans*	http://metazoa.ensembl.org/Caenorhabditis_elegans/Info/Index
	Strongyloides ratti	http://metazoa.ensembl.org/Strongyloides_ratti/Info/Index
	Brugia malayi	http://metazoa.ensembl.org/Brugia_malayi/Info/Index
Mollusca	*Lottia gigantea*	http://metazoa.ensembl.org/Lottia_gigantea/Info/Index
	Octopus bimaculoides	http://metazoa.ensembl.org/Octopus_bimaculoides/Info/Index
	Crassostrea gigas	http://metazoa.ensembl.org/Crassostrea_gigas/Info/Index
Annelida	*Capitella teleta*	http://metazoa.ensembl.org/Capitella_teleta/Info/Index
	Helobdella robusta	http://metazoa.ensembl.org/Helobdella_robusta/Info/Index
Arthropoda	*Apis mellifera*	http://metazoa.ensembl.org/Apis_mellifera/Info/Index
	Bombyx mori	https://silkdb.bioinfotoolkits.net/base/download/-1
	Drosophila melanogaster	http://metazoa.ensembl.org/Drosophila_melanogaster/Info/Index
	Danaus pulex	http://metazoa.ensembl.org/Danaus_plexippus/Info/Index

Echinodermata	*Strongylocentrotus purpuratus*	http://metazoa.ensembl.org/Strongylocentrotus_purpuratus/Info/Index
Fish	*Latimeria chalumnae*	http://asia.ensembl.org/Latimeria_chalumnae/Info/Index
	Branchiostoma floridae	https://www.ncbi.nlm.nih.gov/genome/?term=Branchiostoma_floridae
	Danio rerio	http://asia.ensembl.org/Danio_rerio/Info/Index
Amphibian	*Xenopus tropicalis*	http://asia.ensembl.org/Xenopus_tropicalis/Info/Index
	Nanorana parkeri	https://www.ncbi.nlm.nih.gov/genome/?term=Nanorana_parkeri
Reptilia	*Anolis carolinensis*	http://asia.ensembl.org/Anolis_carolinensis/Info/Index
	Pelodiscus sinensis	http://asia.ensembl.org/Pelodiscus_sinensis/Info/Index
	Alligator sinensis	https://www.ncbi.nlm.nih.gov/genome/?term=Alligator_sinensis%5D
Aves	*Gallus gallus*	http://asia.ensembl.org/Gallus_gallus/Info/Index
	Pseudopodoces humilis	https://www.ncbi.nlm.nih.gov/genome/?term=Pseudopodoces_humilis
	Struthio camelus	https://www.ncbi.nlm.nih.gov/genome/?term=Struthio_camelus
Mammalia	*Ornithorhynchus anatinus*	http://asia.ensembl.org/Ornithorhynchus_anatinus/Info/Index
	Ovis aries	http://asia.ensembl.org/Ovis_aries/Info/Index
	Homo sapiens	http://asia.ensembl.org/Homo_sapiens/Info/Index
