# Supplementary material for: Genome-wide analysis of DNA G-quadruplex motifs across 37 species provides insights into G4 evolution
Source: Commun Biol. 2021 Jan 22;4:98. doi: 10.1038/s42003-020-01643-4 (PMC7822830; doi:10.1038/s42003-020-01643-4)
Supplement: Supplementary file 3 — Description of Additional Supplementary Files [file 42003_2020_1643_MOESM3_ESM.pdf]

Description of Additional Supplementary Files

File Name: Supplementary Data 1
Description: Source data underlying plots shown in figures.

File Name: Supplementary Data 2
Description: Results of functional enrichment of the genes bearing $(G/C)_3L_{1-7}$ motifs in the upstream 2kb regulatory region in 19 representative species.
