# Supplementary material for: Genome-wide analysis of DNA G-quadruplex motifs across 37 species provides insights into G4 evolution
Source: Commun Biol. 2021 Jan 22;4:98. doi: 10.1038/s42003-020-01643-4 (PMC7822830; doi:10.1038/s42003-020-01643-4)
Supplement: Supplementary file 6 — Reporting Summary [file 42003_2020_1643_MOESM6_ESM.pdf]

nature research

Corresponding author(s): Qili Feng; Hui Xiang; Qisheng Song

Last updated by author(s): Nov 23, 2020

Reporting Summary

Nature Research wishes to improve the reproducibility of the work that we publish. This form provides structure for consistency and transparency in reporting. For further information on Nature Research policies, see Authors & Referees and the Editorial Policy Checklist .

Statistics

For all statistical analyses, confirm that the following items are present in the figure legend, table legend, main text, or Methods section.

n/a	Confirmed	
☐	☒	The exact sample size (*n*) for each experimental group/condition, given as a discrete number and unit of measurement
☐	☒	A statement on whether measurements were taken from distinct samples or whether the same sample was measured repeatedly
☐	☒	The statistical test(s) used AND whether they are one- or two-sided<br>*Only common tests should be described solely by name; describe more complex techniques in the Methods section.*
☒	☐	A description of all covariates tested
☒	☐	A description of any assumptions or corrections, such as tests of normality and adjustment for multiple comparisons
☐	☒	A full description of the statistical parameters including central tendency (e.g. means) or other basic estimates (e.g. regression coefficient) AND variation (e.g. standard deviation) or associated estimates of uncertainty (e.g. confidence intervals)
☒	☐	For null hypothesis testing, the test statistic (e.g. *F*, *t*, *r*) with confidence intervals, effect sizes, degrees of freedom and *P* value noted<br>*Give P values as exact values whenever suitable.*
☒	☐	For Bayesian analysis, information on the choice of priors and Markov chain Monte Carlo settings
☒	☐	For hierarchical and complex designs, identification of the appropriate level for tests and full reporting of outcomes
☒	☐	Estimates of effect sizes (e.g. Cohen's *d*, Pearson's *r*), indicating how they were calculated

Our web collection on statistics for biologists contains articles on many of the points above.

Software and code

Policy information about availability of computer code

Data collection	Data were downloaded by fileZilla(3.45.1)
Data analysis	G4 motif sequences were predicted by quadparser version 2.0. Statistical analyses were generated by IBM SPSS Statistics 22.

For manuscripts utilizing custom algorithms or software that are central to the research but not yet described in published literature, software must be made available to editors/reviewers. We strongly encourage code deposition in a community repository (e.g. GitHub). See the Nature Research guidelines for submitting code & software for further information.

Data

Policy information about availability of data

All manuscripts must include a data availability statement. This statement should provide the following information, where applicable:

- Accession codes, unique identifiers, or web links for publicly available datasets
- A list of figures that have associated raw data
- A description of any restrictions on data availability

Links of genome and annotation data:
Saccharomyces cerevisiae http://fungi.ensembl.org/Saccharomyces_cerevisiae/Info/Index
Plasmodium reichenowi http://protists.ensembl.org/Plasmodium_reichenowi_gca_001601855/Info/Index
Paramecium tetraurelia http://protists.ensembl.org/Paramecium_tetraurelia/Info/Index
Dictyostelium discoideum http://protists.ensembl.org/Dictyostelium_discoideum/Info/Index
Schistosoma mansoni http://metazoa.ensembl.org/Schistosoma_mansoni/Info/Index
Macrostomum lignano https://www.ncbi.nlm.nih.gov/genome/?term=Macrostomum_lignano
Echinococcus granulosus https://www.ncbi.nlm.nih.gov/genome/?term=Echinococcus_granulosus
Nematostella vectensis http://metazoa.ensembl.org/Nematostella_vectensis/Info/Index

Acropora digitifera https://www.ncbi.nlm.nih.gov/genome/?term=Acropora_digitifera
Hydra vulgaris https://www.ncbi.nlm.nih.gov/genome/?term=Hydra_vulgaris
Caenorhabditis elegans http://metazoa.ensembl.org/Caenorhabditis_elegans/Info/Index
Strongyloides ratti http://metazoa.ensembl.org/Strongyloides_ratti/Info/Index
Brugia malayi http://metazoa.ensembl.org/Brugia_malayi/Info/Index
Lottia gigantea http://metazoa.ensembl.org/Lottia_gigantea/Info/Index
Octopus bimaculoides http://metazoa.ensembl.org/Octopus_bimaculoides/Info/Index
Crassostrea gigas http://metazoa.ensembl.org/Crassostrea_gigas/Info/Index
Capitella teleta http://metazoa.ensembl.org/Capitella_teleta/Info/Index
Helobdella robusta http://metazoa.ensembl.org/Helobdella_robusta/Info/Index
Apis mellifera http://metazoa.ensembl.org/Apis_mellifera/Info/Index
Bombyx mori http://silkworm.genomics.org.cn/silkdb/#
Drosophila melanogaster http://metazoa.ensembl.org/Drosophila_melanogaster/Info/Index
Danaus pulex http://metazoa.ensembl.org/Danaus_plexippus/Info/Index
Strongylocentrotus purpuratus http://metazoa.ensembl.org/Strongylocentrotus_purpuratus/Info/Index
Latimeria chalumnae http://asia.ensembl.org/Latimeria_chalumnae/Info/Index
Branchiostoma floridae https://www.ncbi.nlm.nih.gov/genome/?term=Branchiostoma_floridae
Danio rerio http://asia.ensembl.org/Danio_rerio/Info/Index
Xenopus tropicalis http://asia.ensembl.org/Xenopus_tropicalis/Info/Index
Nanorana parkeri https://www.ncbi.nlm.nih.gov/genome/?term=Nanorana_parkeri
Anolis carolinensis http://asia.ensembl.org/Anolis_carolinensis/Info/Index
Pelodiscus sinensis http://asia.ensembl.org/Pelodiscus_sinensis/Info/Index
Alligator sinensis https://www.ncbi.nlm.nih.gov/genome/?term=Alligator_sinensis%5D
Gallus gallus http://asia.ensembl.org/Gallus_gallus/Info/Index
Pseudopodoces humilis https://www.ncbi.nlm.nih.gov/genome/?term=Pseudopodoces_humilis
Struthio camelus https://www.ncbi.nlm.nih.gov/genome/?term=Struthio_camelus
Ornithorhynchus anatinus http://asia.ensembl.org/Ornithorhynchus_anatinus/Info/Index
Ovis aries http://asia.ensembl.org/Ovis_aries/Info/Index
Homo sapiens http://asia.ensembl.org/Homo_sapiens/Info/Index
Go annotation data:
http://asia.ensembl.org/biomart/martview/a711e156a54e647e61290eadf58122a5

Field-specific reporting

Please select the one below that is the best fit for your research. If you are not sure, read the appropriate sections before making your selection.

☒ Life sciences ☐ Behavioural & social sciences ☐ Ecological, evolutionary & environmental sciences

For a reference copy of the document with all sections, see nature.com/documents/nr-reporting-summary-flat.pdf

Life sciences study design

All studies must disclose on these points even when the disclosure is negative.

Sample size	Specific sample sizes are described in figures or figure legends. The sample size used for experiment is based on previous experience from the Xiang and Feng labs. No statistical test was used to pre-determine sample size.
Data exclusions	No samples or animals were excluded from the analyses.
Replication	The number of repeats for each experiments are described in corresponding figure legends. All repeats support the same conclusion.
Randomization	Cells or animal tissue were randomly assigned to groups.
Blinding	No, we were not blinded to group allocation. The results are from the bioinfomatic analyses on the resequencing data, which are not a bit influenced by personal mind.

Reporting for specific materials, systems and methods

We require information from authors about some types of materials, experimental systems and methods used in many studies. Here, indicate whether each material, system or method listed is relevant to your study. If you are not sure if a list item applies to your research, read the appropriate section before selecting a response.

Materials & experimental systems

n/a	Involved in the study	
☐	☒	Antibodies
☐	☒	Eukaryotic cell lines
☒	☐	Palaeontology
☐	☒	Animals and other organisms
☒	☐	Human research participants
☒	☐	Clinical data

Methods

n/a	Involved in the study	
☒	☐	ChIP-seq
☒	☐	Flow cytometry
☒	☐	MRI-based neuroimaging

Antibodies

Antibodies used	BG4 (Ab00174-1.6, Absolute antibody,Oxford, UK) ; anti-His antibody (B1023, Beijing Biodragon ImmunoTechnologies, Beijing, China) ; anti-rabbit Alexa 594-conjugated (A11037, Invitrogen, CA, USA)
Validation	BG4: This chimeric mouse antibody Fab-fragment was made using the variable domain sequences of the original Human scFv format, for improved compatibility with existing reagents, assays and techniques. It does not have a FLAG-tag, but a His-tag, which may be used for detection.<br>anti-His antibody: The antigen of this antibody is synthetic peptide corresponding to residues H H H H H H. This antibody were produced in rabbit.

Eukaryotic cell lines

Policy information about cell lines

Cell line source(s)	Drosophila melanogaster KC cells, Gallus gallus DF-1 cells and Homo sapiens LO2 cells were obtained from the American Type Culture Collection. Ovis aries OAR-L1 cells were provided by Kunming Instituted of Zoology.
Authentication	KC, DF-1, OAR-L1 and LO2 cells were authenticated based on our experience working with these cell lines, such as cell morphology, culture conditions, etc.
Mycoplasma contamination	Cells were routinely tested for mycoplasma contamination, and only negative cells were used in experiments.
Commonly misidentified lines (See ICLAC register)	None of the cell lines used are listed in the database of commonly misidentified cell lines maintained by ICLAC.

Animals and other organisms

Policy information about studies involving animals; ARRIVE guidelines recommended for reporting animal research

Laboratory animals	The adult fish of Danio rerio (male) were purchased from China Zebrafish Resource Center (CZRC Catalog ID: CF1), and were used to generate Danio rerio cells. The Pelodiscus sinensis were purchased from Pelodiscus sinensis farmers, and were used to generate Pelodiscus sinensis cells.
Wild animals	The study did not involve samples collected from wild animals.
Field-collected samples	The study did not involve samples collected from field.
Ethics oversight	All animal protocols were approved by Institutional Animal Care and Use Committee (ACUP) at the University of Chicago.

Note that full information on the approval of the study protocol must also be provided in the manuscript.
